# Microgastrinae (Hymenoptera: Braconidae) in the Forest State of Artikutza (Navarra: Spain): Diversity and Community Structure

**DOI:** 10.3390/insects4030493

**Published:** 2013-09-18

**Authors:** Jesica Pérez-Rodríguez, Teresa Oltra-Moscardó, Francisco Javier Peris-Felipo, Ricardo Jiménez-Peydró

**Affiliations:** Laboratory of Entomology and Pest Control, Institute Cavanilles of Biodiversity and Evolutionary Biology, University of Valencia, c/. Catedrático José Beltrán, 2, 46980 Paterna, Valencia, Spain; E-Mails: jepero@alumni.uv.es (J.P.-R.); Teresa.oltra@uv.es (T.O.-M.); Ricardo.jimenez@uv.es (R.J.-P.)

**Keywords:** Braconidae, Microgastrinae, diversity, community, western Pyrenees, Spain

## Abstract

Microgastrinae is one of the largest subfamilies of the Braconidae with about 2,000 described species worldwide. These wasps are of enormous ecological interest due to their role in controlling the caterpillar populations. This study analyses diversity and community structure within the Microgastrinae in the Artikutza Forest, located in the Peñas de Aia Natural Park, western Pyrenees, Spain. The specimens were collected in two different habitats: mixed forest and beech forest. A total of 524 specimens, belonging to nine separate genera and 27 species were captured. Alpha, beta and gamma diversity were analyzed. Additionally, the relationship between Microgastrinae phenology and climatic conditions were studied.

## 1. Introduction

Parasitic Hymenoptera are extremely species-rich and of wide significance in terrestrial ecosystems, because they are a major group of insects with a parasitoid lifestyle [1]. The fact that they are particularly sensitive to environmental disturbance makes them good indicators of diversity and environmental stability [1,2] and allows the establishment of a pattern of ecosystem conservation [3,4,5].

Within the parasitic Hymenoptera, the family of Braconidae is the second largest family, comprising about 40,000 species, which are distributed throughout the world in several different habitats [6]. They are considered essential for the maintenance of the balance of the communities that include them [7], due to their role in controlling the caterpillar populations [8,9,10,11]. These wasps are primary parasitoids of the immature stages of Lepidoptera, Diptera and Coleoptera [6,12] and the economic importance of braconid species resides in their potential for the biological control of insect pests [8,13,14].

Within the Braconidae, the subfamily Microgastrinae with over 2,000 species described and an estimated global diversity of 5,000–10,000 [15], is one of the most important groups of parasitoids in terms of both species richness and economic importance [16]. This fact has caused the Microgastrinae to be one of the most studied parasitic wasps by DNA-barcode in recent years [16,17,18,19].

These wasps are all koinobiont endoparasitoids of Lepidopteran larvae and the vast majority of known hosts belong to the *Ditrysia*-group [2]. More than 100 species of Microgastrinae have been used in the biological control of Lepidoptera pests and this total is likely to rise [20]. 

Despite considerable knowledge concerning the ecology of some species, the same is not true at the family level with the exception of some recent studies [21,22]. In the Iberian Peninsula (Andorra, Spain and Portugal), Braconidae communities have been insufficiently analyzed [4,23,24,25,26,27,28], with the exception of those in the Pyrenees [29] or in Navarra [30,31].

Within this context, this study analyzed alpha, beta and gamma diversity of Microgastrinae in the Artikutza Forest (Navarra) located in the western Pyrenees and having an enormous ecological value. This area includes two adjacent types of forest delimited by a stream: mixed pine forest and beech forest. Data on the phenology of the subfamily and its relationship with environmental and climatic conditions were also studied.

## 2. Experimental Section

### 2.1. Study Area

Sample collection took place in the Artikutza Estate (30TWN972868 U.T.M), located within the Natural Park Peñas de Aia (Guipúzcoa, Spain), in the western Pyrenees, having an extent of 5 ha and an altitude between 575 and 652 m [4]. Since its creation, the estate has gone through frequent processes of deforestation and repopulation, but is currently populated by two adjacent plant habitats: mixed forest and beech forest. The mixed forest is about 70 years old and is dominated by secondary stands of *Pinus sylvestris* L., *Quercus petraea* L. and *Fagus sylvatica* L. The beech forest is of similar age, has been only partially repopulated and is surrounded by conifer plantations. Apart from beech, there are isolated specimens of *Castanea sativa* L., *Taxus baccata* L., *Salix atrocinerea* Brot., *Fraxinus excelsior* L., *Betula celtiberica* Rothm & Vasc., *Ulmus glabra* Hud. and *Sorbus aucuparia* (L.) Crantz. The oligotrophic nature of the soil produces a poor shrubbery and herbaceous stratum, in which the following species commonly occur: *Ilex aquifolium* L., *Blechnum spicant* (L.) Roth., *Deschampsia flexuosa* (L.) Trin., *Vaccinium myrtilus* L., *Euphorbia amygdaloides* L., *Daphne laureola* L., *Oxalis acetosella* L. and in the brighter area and clearing edges, *Crataegus monogyna* Jacq., *Corylus avellana* Miller, *Pyrus cordata* Desv., *Malus sylvestris* Miller, *Pteridium aquilinum* (L.) Kuhn., *Erica vagans* L. and *Asphodelus albus* Miller, among others. The main climatic feature of the area is the high precipitation regime, between 1,500 and 2,800 mm. Rainfall is more abundant in winter and scarcer during summer. The temperature is moderate, with a mean between 8 and 17 °C. 

To identify Microgastrinae genera, Mason [32] and Whitfield [20] criteria were followed. Species were determined by the keys of Papp [33,34,35,36,37,38,39,40,41,42,43,44,45,46,47] and Tobias *et al.* [48] that were made for females because it is difficult to identify species with males due to the similarity between males of different genera. The specimens are deposited in the Entomological collection of University of Valencia (ENV).

### 2.2. Sampling Design

Sampling was carried out using Malaise traps, which are one of the best systems to capture flying insects passively. In this study, the bidirectional Townes model was used. Collected specimens were conserved in a jar of preserving liquid comprising 75% alcohol, 5% acetic acid and 20% distilled water.

Six Malaise traps were placed in total: three in the mixed forest (M-1, M-2 and M-3) and three in the beech forest (H-1, H-2 and H-3). The hill orientation, orientation with respect to the North Pole, capture orientation, altitude and trap gradient are shown in Table 1. The heterogeneous distribution of trees and the differences in slope gave each trap specific conditions, the main features of which were as follows:

M-1: On the edge of a clearing.
M-2: In open forest.
M-3: Under a mature beech tree in an area mostly populated with pine trees.
H-1: Under a large beech tree.
H-2: Near a small clearing and surrounded by a copse of beech shrubs.
H-3: Under a shade of a mature beech tree on a landing.

Sampling was performed without interruption during a two-year period, from May 1995 to April 1997 and produced a total of 46 samples per trap in 733 days (25 and 21 samples over 362 and 371 days, respectively). During this period, jars were collected at intervals of 14 days with the exception of seven occasions, when they were collected after 28 days, to give a total of 270 samples.

**Table 1:** Location of the traps situated in mixed and beech forest (Artikutza, Navarra).

Trap	Hill orientation	North Pole orientation	Axis trap orientation	Altitude	Hill gradient
M-1	NE-SW	N230E	N-S	611 m	12°
M-2	NE-SW	N210E	N-S	631 m	15°
M-3	NE-SW	N216E	N-S	652 m	20°
H-1	NW-SW	N235E	N-S	576 m	19°
H-2	NW-SW	N210E	N-S	595 m	18°
H-3	NW-SW	N242E	N-S	620 m	9°

Meteorological data were recorded by the meteorological station located in the village of Artikutza. Rainfall during the sampling period was 1,778 mm for the first year and 2,190 mm for the second year. The rainiest months were February and November 1996 (372 and 636 mm, respectively) and October, June and March 1995 (23, 29 and 55 mm, respectively) were the driest. Temperature remained in a moderate range, with a minimum monthly mean of 5 °C for February 1996 and a maximum of 20 °C for July 1995. The second sampling period was colder and rainier than the first, particularly during the summer and autumn months. As well as analysing the relationship between climatic conditions and specimens captured, the phenology of Microgastrinae was also studied. 

### 2.3. Diversity and Community Structure

Once the Microgastrinae specimens had been identified, alpha, beta and gamma biodiversity indices for each trap and habitat were calculated. Alpha diversity is the richness of species in a homogeneous community and was measured by taxa richness, abundance and dominance. Taxa richness was used for evaluating the richness of sampling areas. This was calculated by the Margalef index, which transforms the number of species per sample into the proportion to which the species are added by expansion of the sample, and establishes a functional relationship between number of species and the total number of specimens [49]. Abundance is a concept used for evaluating faunal composition within a given area [50]. This was calculated using the Shannon index, because it measures equity, indicating the degree of uniformity in species representation (in order of abundance), while considering all samples [49,50,51].

Dominance is negative and represents equity [50] and it takes into account the representativeness of the species with the highest value of importance, without assessing the contribution from the rest of the species. Dominance was calculated using the Simpson index. 

Beta diversity is the degree of change or substitution in species composition between different communities within the same landscape. To measure beta diversity, the Jaccard and Complementarity indices were used. The Jaccard index relates the total number of species shared between two sites to the total number that is found in only one of the sites, *i.e.*, exclusive species. It is a qualitative coefficient, the range of which varies from 0 when no species are shared between both sites to 1 when both sites have an identical species composition and abundance [49,51]. The Complementarity index indicates the degree of similarity in species composition and abundance between two or more communities [49,51].

Gamma diversity was calculated using the Lande index and indicates the diversity value of all environments under study, as expressed by the richness indices for each area (alpha diversity) and the difference between them (beta diversity) [51,52]. 

The Microgastrinae community structure in mixed and beech forest was finally analysed by *log-series*, *log-normal* and *broken-stick* models [50]. The log-series model represents a community composed of a few abundant species and a large number of rare species. The *broken-stick* model refers to the maximum occupation of an environment with equitable sharing of resources between species and the *log-normal* model reflects an intermediate situation between the two [53]. Each of these models was applied to data to test their significance, together with chi-squared analysis [54].

## 3. Results and Discussion

During the sampling period, 3,534 specimens of the Braconidae family were captured. Of these, 524 belonged to the Microgastrinae subfamily (14.82%), 364 of which were males and 160 were females. Because only females could be identified at the species level, males were not included in the analyses. From the total of 160 females, 154 could be identified because the remaining specimens lacked some structure that was essential for their identification. 

A total of 27 species was captured, belonging to nine genera: *Choeras dorsalis* (Spinola 1808), *Choeras hedymeles* (Nixon 1973), *Choeras parasitellae* (Bounché 1834), *Cotesia ancilla* (Nixon 1974), *Cotesia chares* (Nixon 1974), *Cotesia jucundus* (Marshall 1885), *Cotesia* sp1, *Dolichogenidea celsus* (Papp 1975), *Dolichogenidea laevigatoides* (Nixon 1972), *Dolichogenidea laevigatus* (Ratzeburg 1848), *Dolichogenidea* sp1, *Dolichogenidea* sp2, *Dolichogenidea varifemur* (Abdinbekova 1969), *Glyptapanteles aliphera* (Nixon 1973), *Glyptapanteles fulvipes* (Haliday 1834), *Glyptapanteles mygdonia* (Nixon 1973), *Glyptapanteles porthetriae* (Muesebeck 1928), *Glyptapanteles vitripennis* (Curtis 1830), *Microgaster* sp1, *Microplitis marshalii* Kokijev 1898, *Paroplitis* sp1, *Paroplitis wesmaeli* (Ruthe 1860), *Pholetesor circumscriptus* (Nees 1834), *Pholetesor* sp1, *Pholetesor* sp2, *Protapanteles anchisiades* (Nixon 1973) and *Protapanteles* sp1.

The number of genera and species differed between each habitat and trap. In the mixed forest, 23 species were collected (15 in M-1, 8 in M-2 and 10 in M-3) whereas 11 species were captured in the beech forest (7 in H-1, 7 in H-2 and 7 in H-3) (Table 2). These differences are because the mixed forest represents an area with a richer diversity of plants and consequently also of host species. Individually, M-1 was the trap with the largest number of species as it was located on the edge of a clearing which was windier than other locations.

The genus analysis showed that the genus *Choeras* is the most abundant in this study, with 64 specimens, followed by the genera *Paroplitis* (45) and *Glyptapanteles* (35) which contrasts with the results from other studies of Braconidae in the Iberian Peninsula [55]. When these results are checked with the results obtained by Falcó-Garí *et al.* in Andorra [29], it is possible to see that *Glyptapanteles* remains very abundant. However, only one specimen has been captured of *Microplitis*. 

The species analysis showed that *Choeras hedymeles* was the most common species with 59 specimens (38.31%) followed by *Glyptapanteles vitripennis* with 21 and *Dolichogenidea* sp1 with 15 (13.63% and 9.74%, respectively). However, analysis of the number of captures showed that 102 specimens were collected in the mixed forest habitat (37 in M-1, 28 in M-2 and 37 in M-3) and 52 in the beech forest (18 in H-1, 18 in H-2 and 16 in H-3) (Table 2). Note the absence of the genus *Apanteles*, genus characterized by its ubiquity and dominance [56]. However, checking the data capture of Falcó-Garí *et al.* [29], in Andorra (Pyrenees) also note that only 15 specimens were captured of a total of 494 (3.03%). Both facts show that *Apanteles* is not abundant in this area.

**Figure 1:**
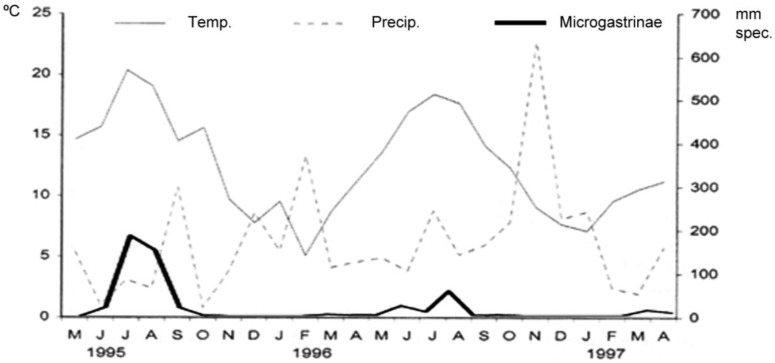
Relationship between climatic conditions and abundance of Microgastrinae.

The phenology of the Microgastrinae (Figure 1) established relationships between the abundance of Microgastrinae and climatic conditions. The capture of Microgastrinae was higher during the summer months when temperatures are moderate with average of 15–20 °C. In contrast, increased rainfall during the winter months caused a decrease in temperature and in Microgastrinae abundance.

**Table 2:** Distribution of Microgastrinae specimens per habitat.

Species	Trap number						Total Specimens		
	M-1	M-2	M-3	H-1	H-2	H-3	ΣM	ΣH	Total
*Choeras dorsalis*	1	0	0	0	0	0	1	0	1
*Choeras hedymeles*	5	10	18	10	10	6	33	26	59
*Choeras parasitellae*	3	0	0	0	0	1	3	1	4
*Cotesia ancilla*	1	0	0	0	0	0	1	0	1
*Cotesia chares*	0	1	0	0	0	0	1	0	1
*Cotesia jucundus*	0	1	0	0	0	0	1	0	1
*Cotesia* sp1	4	0	0	0	0	0	4	0	4
*Dolichogenidea celsus*	0	0	0	1	0	0	0	1	1
*Dolichogenidea laevigatoides*	0	0	0	2	0	0	0	2	2
*Dolichogenidea laevigatus*	0	0	0	1	1	0	0	2	2
*Dolichogenidea* sp1	3	3	5	2	2	0	11	4	15
*Dolichogenidea* sp2	0	0	1	0	0	0	1	0	1
*Dolichogenidea varifemur*	0	0	0	0	1	1	0	2	2
*Glyptapanteles aliphera*	1	0	0	0	0	0	1	0	1
*Glyptapanteles fulvipes*	2	0	0	0	0	0	2	0	2
*Glyptapanteles mygdonia*	0	1	3	1	0	4	4	5	9
*Glyptapanteles porthetriae*	2	0	0	0	0	0	2	0	2
*Glyptapanteles vitripennis*	4	9	4	1	2	1	17	4	21
*Microgaster* sp1	1	0	0	0	0	0	1	0	1
*Microplitis marshalii*	1	0	0	0	0	0	1	0	1
*Paroplitis macrocephalus*	0	0	1	0	0	0	1	0	1
*Paroplitis wesmaeli*	1	0	0	0	1	2	1	3	4
*Pholetesor circumscriptus*	0	0	1	0	0	0	1	0	1
*Pholetesor* sp1	3	0	1	0	1	1	4	2	6
*Pholetesor* sp2	0	0	1	0	0	0	1	0	1
*Protapanteles anchisiades*	5	2	2	0	0	0	9	0	9
*Protapanteles* sp1	0	1	0	0	0	0	1	0	1
*Total specimens*	37	28	37	18	18	16	102	52	154

### 3.1. Alpha Diversity

The mixed forest had a higher species richness with a D*_Mg_* = 4.76 than the beech forest with a value of 2.53. At the trap level, M-1 (D*_Mg_* = 3.877) had the highest richness, followed by H-3 (2.164) and H‑1 and H-2 (1.750 and 1.650, respectively) (Table 3). In addition to alpha diversity, the Shannon–Wiener and the Simpson indexes are shown in Table 3. The Shannon index for mixed forest (2.346) and beech forest (2.017) suggested a similar trend in the distribution of dominant genera and discrepancies were due to different numbers of rare genera (those represented by few specimens). This trend was also supported by the Simpson index (0.159 in mixed forest and 0.281 in beech forest). At the trap level, the Shannon-Wiener index values were higher in M-1, M-3 and H-3 (2.537, 1.711 and 1.667, respectively) whereas for the Simpson index, the ranking was H-1 and H-2 (both 0.345) M-2 (0.525) and M-3 (0.280). 

**Table 3:** Diversity and abundance values of the collected Microgastrinae species.

Data	Trap number						Total	
	M-1	M-2	M-3	H-1	H-2	H-3	ΣM	ΣH
Species	15	8	10	7	7	7	23	11
Specimens	37	28	37	18	18	16	102	52
Shannon I.	2.537	1.636	1.711	1.453	1.453	1.667	2.346	2.174
Simpson I.	0.090	0.252	0.280	0.345	0.345	0.234	0.159	0.281
Margalef I.	3.877	2.101	2.492	2.076	2.076	2.164	4.760	2.530

### 3.2. Beta Diversity

Beta diversity was calculated using values between the different areas with the Jaccard and complementarity indices (Table 4). Firstly, the Jaccard index showed a degree of dissimilarity at the species level between the two habitats (0.259). At the trap level, H-2 and H-3 both showed the highest similarity with each other (0.556) and M-1 and H-1 were the least similar traps (0.158). Secondly, the complementarity index between mixed and beech forest (0.741) indicated that the species composition similarity between both communities was low. At the trap level, M-1 and H-1 shows the highest complementarity (0.842) whereas the comparison between H-2 and H-3 showed the least complementarity, with a value of 0.444.

**Table 4:** Jaccard index and Complementarity Values.

	M-1	M-2	M-3	H-1	H-2	H-3	
M-1		0.211	0.250	0.158	0.294	0.294	
M-2	0.789		0.385	0.364	0.250	0.250	
M-3	0.750	0.615		0.308	0.308	0.308	Jaccard I.
H-1	0.842	0.636	0.818		0.400	0.273	
H-2	0.706	0.750	0.818	0.600		0.556	
H-3	0.706	0.750	0.818	0.545	0.444		
	Complementarity						

### 3.3. Gamma Diversity

Gamma diversity using the Lande index reached a value of 37 and can be decomposed into alpha and beta diversity. Alpha diversity contributed 45.95% towards the gamma index and beta diversity, the remaining 54.05%. These results suggest that a greater degree of change in species composition than differences in species richness exists between mixed and beech communities. This fact can be explained by the specificity of habitat in both potential hosts and their parasitoids.

### 3.4. Community Structure

The study of the Microgastrinae community structure in mixed and beech forest showed that the data in both cases fitted a *log-series* model. For mixed forest, *P* = 0.111 (Table 5) and for beech forest, *P* = 0.269 (Table 6). Both values were greater than the 0.05 significance level and thus indicated an unstable community composed of a few abundant species and a large number of rare species. In conclusion, the mixed forest, with a greater variety of potential host plants of Lepidoptera, showed a higher diversity of Microgastrinae and a greater number of captured specimens. Moreover, the studied communities were composed of a few abundant species and a large number of rare species; in certain weather conditions. 

**Table 5:** Observed and expected frequencies of species according to *log-series*, *log-normal* and *broken-stick* models of Microgastrinae in the mixed forest community.

Mixed forest	Log-series		Log-normal		Broken-stick	
Class	Exp freq.	Obs freq.	Exp freq.	Obs freq.	Exp freq.	Obs freq.
0	-	-	3.41	0	-	-
1	12.35	15	16.93	15	7.63	15
2	4.01	4	4.01	4	4.79	4
3	3.41	0	1.65	0	5.12	0
4	2.19	2	0.36	2	2.91	2
5	0.87	1	0.04	1	0.47	1
6	0.15	1	0	1	0.01	1
	*χ^2^* = 8.963	*P* = 0.111	*χ^2^* = 1031.5	*P* = 1.399·10^−219^	*χ^2^* = 211.26	*P* = 1.104·10^−43^

**Table 6:** Observed and expected frequencies of species according to *log-series*, *log-normal* and *broken-stick* models of Microgastrinae in the beech forest community.

Beech forest	Log-series		Log-normal		Broken-stick	
Class	Exp freq.	Obs freq.	Exp freq.	Obs freq.	Exp freq.	Obs freq.
0	-	-	0.19	0	-	-
1	5.78	6	6.38	6	3.26	6
2	1.90	3	3.05	3	2.27	3
3	1.66	1	1.31	1	8.46	1
4	1.11	0	0.24	0	16.92	0
5	0.478	1	0.02	1	33.85	1
	*χ^2^* = 5.188	*P* = 0.269	*χ^2^* =56.343	*P* = 6.91·10^−11^	*χ^2^* = 57.91	*P* = 7.96·10^−12^

## 4. Conclusions

To sum up, on checking with studies carried out in Artikutza about Braconidae subfamilies, it has been shown that Alysiinae was the most abundant subfamily captured with approximately 64.23%, followed by Microgastrinae with 14.82%, while Opiinae has only 2.97%. The information about the Braconidae abundance and alpha diversity is very interesting due to the specific relationships that these parasitic wasps have with their host and with the host plants. This information about trophic relationships could be used indirectly to further knowledge concerning the biodiversity appearing in similar areas.

On the other hand, it is possible to consider that Microgastrinae is one of the most abundant subfamilies in both the mixed forest and in the beech forest with 524 collected specimens, mainly collected during the months of moderate temperatures (15–20 °C), July and August. In turn, as can be expected, the mixed forest shows a higher Microgastrinae abundance than in the beech forest due to the greater variety of potential host plants of Lepidoptera. However, in contrast to the results from other studies of Braconidae in the Iberian Peninsula, *Choeras* is the most abundant genus with the unexpected absence of *Apanteles*. 

In addition, on analyzing the community structure, it is possible to see that Microgastrinae community of mixed and beech forest are composed of a few abundant species and a large number of rare species. The observed pattern in beta diversity shows that the two sampled sites have a higher complementarity with different species composition, which belongs to areas with an important ecological value.

Finally, we conclude that, although this study was conducted to determine the diversity and community structure of Microgastrinae, further studies of Braconidae are recommended in different areas together with DNA-barcode studies to increase the knowledge of this large subfamily that remains mostly unknown.
